# Supplementary material for: Clinical Significance of Timing of Intubation in Critically Ill Patients with COVID-19: A Multi-Center Retrospective Study
Source: J Clin Med. 2020 Sep 2;9(9):2847. doi: 10.3390/jcm9092847 (PMC7564702; doi:10.3390/jcm9092847)
Supplement: Supplementary file 1 [file jcm-09-02847-s001.pdf]

Supplementary Table S1. Demographics and baseline characteristics of initially nonintubated patients

	Initially Nonintubated ($N = 24$)		P Value
	Never Intubated ($N = 8$)	Late Intubation ($N = 16$)	
Age	72 (63–78)	66 (59–77)	0.520
Male	4 (50)	10 (62.5)	0.673
Body mass index, kg/m^2	26.1 (21.7–26.7)	25.1 (22.7–27.3)	0.947
Smoking status			0.325
Never smoker	5 (62.5)	10 (62.5)	
Former smoker	2 (25)	6 (37.5)	
Current smoker	1 (12.5)	0 (0)	
Comorbidities			
Any comorbidities	6 (75)	13 (81.2)	>0.999
Hypertension	3 (37.5)	8 (50)	0.679
Diabetes	1 (12.5)	7 (43.8)	0.189
Chronic kidney disease	0 (0)	2 (12.5)	0.536
Dementia	2 (25)	1 (6.2)	0.249
Cerebrovascular disease	1 (12.5)	1 (6.2)	>0.999
Malignancy	0 (0)	4 (25)	0.262
Cardiovascular disease	2 (25)	2 (12.5)	0.578
Chronic lung disease	0 (0)	1 (6.2)	>0.999
Chronic liver disease	2 (25)	0 (0)	0.101
Duration of symptoms before admission, days	7 (5–14)	5 (3–10)	0.157
Presenting symptoms			
Fever	7 (87.5)	9 (56.2)	0.189
Dyspnea	5 (62.5)	12 (75)	0.647
Cough	6 (75)	8 (50)	0.388
Sputum	4 (50)	6 (37.5)	0.673
Myalgia	1 (12.5)	4 (25)	0.631
Fatigue	1 (12.5)	6 (37.5)	0.352
Diarrhea	3 (37.5)	2 (12.5)	0.289
Vital signs at the time of ICU admission			
Mean arterial pressure, mmHg	93 (87–110)	93 (86–97)	0.902
Heart rate, beats/min	84 (81–106)	92 (74–100)	0.951
Respiratory rate, breaths/min	21 (20–24)	21 (20–29)	0.679
Body temperature, ℃	36.7 (36.3–37.6)	36.9 (36.6–37.4)	0.296

Data are presented as median (interquartile range) or N (%). ICU, intensive care unit.

Supplementary Table S2. Initial laboratory findings of initially nonintubated patients

	Initially Nonintubated (*N* = 24)		*P* Value
	Never Intubated (*N* = 8)	Late Intubation (*N* = 16)	
White blood cells, 10^3/L	7.23 (4.17–9.55)	7.16 (5.9–9.96)	0.582
Neutrophil, %	76.2 (65.4–82.4)	81.9 (79.3–89.5)	0.050
Lymphocyte, %	14.8 (7.6–25)	9.2 (6.8–13.7)	0.159
Hemoglobin, g/dL	13.6 (12.2–14.4)	13.1 (10.9–14.1)	0.581
Hematocrit, %	40.5 (35.4–43.2)	38.7 (31.8–40.6)	0.374
Platelets, 10^3/L	186 (153–286)	220 (156–295)	0.582
C–reactive protein, mg/dL	7.35 (4.34–12.18)	11 (7.39–17.43)	0.221
Procalcitonin, mmol/L	0.11 (0.08–0.39)	0.13 (0.1–0.21)	0.685
Lactate, mmol/L	1.5 (1–1.8)	1.8 (1.3–2.4)	0.185
Albumin, g/dL	3.4 (3.1–3.5)	3.4 (3.3–3.7)	0.877
AST, U/L	67 (41–94)	50 (33–65)	0.519
ALT, U/L	20 (11–62)	22 (13–53)	0.736
Total bilirubin, mg/dL	0.5 (0.38–1.04)	0.6 (0.3–0.83)	0.646
BUN, mg/dL	12.4 (6.8–20.2)	16.8 (11.8–35.3)	0.221
Creatinine, mg/dL	0.76 (0.64–1.25)	0.95 (0.7–1.9)	0.327
Sodium, mmol/L	136 (134–137)	137 (132–139)	0.422
Potassium, mmol/L	4.3 (3.2–5)	3.9 (3.3–4.6)	0.690
Glucose, mg/dL	107 (88–135)	161 (122–172)	0.030
LDH, U/L	433 (362–518)	468 (344–698)	0.772
D–dimer, ug/mL	2.76 (1.23–9.95)	2.03 (1.07–3.74)	0.673
Prothrombin time, INR	1.09 (1.01–1.95)	1.08 (1.02–1.27)	0.697
NT-proBNP, pg/mL	599 (139–1,044)	540 (372–2,026)	0.606
Troponin I, ng/mL	0.02 (0.01–0.02)	0.02 (0.01–0.02)	0.968
CK-MB, U/L	1.6 (0.7–2.8)	1 (0.8–1.4)	0.301

Data are presented as median (interquartile range). AST, aspartate aminotransferase; ALT, alanine aminotransferase; BUN, blood urea nitrogen; LDH, lactate dehydrogenase; NT-proBNP, N-terminal probrain natriuretic peptide; CK-MB, creatine kinase-MB.

Supplementary Table S3. Severity of illness and clinical course of initially nonintubated patients

	Initially Nonintubated (*N* = 24)		*P* Value
	Never Intubated (*N* = 8)	Late Intubation (*N* = 16)	
Severity of illness on ICU admission			
Septic shock	1 (12.5)	1 (6.2)	>0.999
Acute kidney injury	1 (12.5)	4 (25)	0.631
Acute cardiac injury	1 (12.5)	2 (12.5)	>0.999
SOFA score	2 (2–4)	3 (2–4)	0.250
APACHE II score	10 (8–11)	14 (8–15)	0.176
ABGA at the time of diagnosis of ARDS			
pH	7.47 (7.45–7.51)	7.43 (7.4–7.49)	0.076
PaCO2, mmHg	32.2 (29.1–34.8)	32.3 (23.8–37.1)	0.783
PaO2, mmHg	63.8 (51.4–111)	67.8 (55.7–79.7)	0.951
HCO3, mmol/L	22.8 (22.1–24.4)	20.8 (17.4–26.3)	0.220
PF ratio	173 (114–238)	120 (62–188)	0.220
ICU management			
HFNC	8 (100)	16 (100)	
NM blockade	0 (0)	9 (56.2)	0.009
CRRT	0 (0)	5 (31.2)	0.130
Tracheostomy	0 (0)	7 (43.8)	0.054
ECMO	0 (0)	4 (25)	0.262
Medical treatment			
Antiviral agents			
Lopinavir-ritonavir	5 (62.5)	11 (68.8)	>0.999
Darunavir-cobicistat	2 (25)	5 (31.2)	>0.999
Antibiotics	8 (100)	16 (100)	
Hydroxychloroquine	8 (100)	14 (87.5)	0.536
Glucocorticoid	4 (50)	15 (93.8)	0.028
Medical event during ICU care			
Septic shock	1 (12.5)	14 (87.5)	0.001
Acute kidney injury	0 (0)	7 (43.8)	0.054
Acute cardiac injury	1 (12.5)	4 (25)	0.631
VAP or HAP	0 (0)	1 (6.2)	>0.999
CRBSI	0 (0)	3 (18.8)	0.526
Bleeding	0 (0)	3 (18.8)	0.526
CPCR	1 (12.5)	2 (12.5)	>0.999

Data are presented as median (interquartile range) or *N* (%). ICU, intensive care unit; SOFA, Sepsis–related Organ Failure Assessment; APACHE II, Acute Physiology and Chronic Health Evaluation; ABGA, arterial blood gas analysis; PF ratio, arterial partial pressure of oxygen (PaO2)/fraction of inspired oxygen (FiO2) ratio; HFNC, high–flow nasal cannula; NM blockade, neuromuscular blockade; CRRT, continuous renal replacement therapy; ECMO, extracorporeal membrane oxygenation; VAP, ventilator–associated pneumonia; HAP, hospital–acquired pneumonia; CRBSI, catheter–related bloodstream infection; CPCR, cardiopulmonary–cerebral resuscitation.
